# Interventions for improving metabolic risk in overweight Latino youth

**DOI:** 10.3109/17477161003770123

**Published:** 2010-10

**Authors:** Jaimie N Davis, Emily E Ventura, Gabriel Q Shaibi, Courtney E Byrd-Williams, Katharine E Alexander, Amanda K Vanni, Mathew R Meija, Marc J Weigensberg, Donna Spruijt-Metz, Michael I Goran

**Affiliations:** 1 Department of Preventive Medicine, Keck School of Medicine, University of Southern CaliforniaUSA; 2 College of Nursing and Healthcare Innovation, Arizona State UniversityUSA; 3 Department of Pediatrics, Keck School of Medicine, University of Southern CaliforniaUSA; 4 Department of Physiology and Biophysics, Keck School of Medicine, University of Southern CaliforniaUSA

**Keywords:** Latino adolescents, randomized controlled trials, insulin sensitivity and secretion, diet and exercise interventions, adiposity

## Abstract

This review highlights various components of interventions that reduced obesity and type 2 diabetes risk factors among overweight Latino youth. A total of 114 overweight Latino adolescents completed one of four randomized controlled trials: 1) strength training (ST; boys only); 2) modified carbohydrate nutrition program (N); 3) combination of N+ST; or 4) N + Combination of Aerobic and ST (N+CAST; girls only). Measures included: strength by 1-repetition max, dietary intake by 3-d records, body composition by DEXA/MRI, glucose/insulin indices by oral and IV glucose tolerance tests. ST improved insulin sensitivity by 45% in Latino boys, and N, N+ST, and N+CAST improved glucose control in Latino boys and girls. The CAST approach reduced all adiposity measures by ∼3% in Latina girls. Participants who decreased added sugar, increased dietary fiber, and had increased parental attendance, regardless of intervention group, improved insulin action and reduced visceral adipose tissue. In conclusion, ST, CAST, and a modified carbohydrate nutrition program with separate parental classes were all successful components of the interventions that decreased obesity and related metabolic diseases.

## Introduction

Latino youth are 1.5–1.7 times more likely to be obese than Caucasian children, with 40.5% and 37.1% of Latino boys and girls, respectively, (aged 12 to 19 years) being overweight (≥85^th^ CDC age- and gender-specific percentile) compared with 34.5% and 31.7%, respectively, of Caucasian children being overweight (1,2). In the Latino population, the increase in pediatric obesity has been closely paralleled by increasing risk for metabolic diseases, such as type 2 diabetes, fatty liver disease and metabolic syndrome (3). Latino children are more likely to be insulin resistant than their Caucasian counterparts, independent of adiposity (4). Our group has shown that over 30% of overweight Latino children (aged 8 to 14 years) in the Los Angeles area have pre-diabetes (5,6) and the metabolic syndrome (7). While there are numerous pediatric interventions aimed at decreasing obesity (8,9), few randomized trials have targeted Latinos or addressed the underlying metabolic abnormalities, specifically visceral fat, insulin secretion and resistance.

One of the few interventions conducted in Latino youth was the Bienstar Program, which was a large school-based, randomized control trial with 3 096 4^th^ grade students, who were 80% Latino and 94% low socioeconomic status. This 7-month intervention focused on decreasing fat intake and increasing dietary fiber and physical activity, resulted in significant reductions in fasting glucose levels (adjusted difference of −2.24 mg/dL) compared with control schools (10). A recent school-based randomized control trial with 60 overweight Latino children (aged 10–14 years), found that an intensive 12-week nutrition and exercise intervention (5x/week [wk]) resulted in a significant 15% reduction in BMI z-scores and a 3% reduction in total cholesterol at 6-month follow-up; however, no significant improvements in glucose or insulin were found (11). Our interventions build on these previous studies by examining the effects of diet and exercise interventions on precise measures of insulin sensitivity/secretion, and beta-cell functioning.

We have previously conducted four randomized, controlled interventions, involving nutrition, physical activity or a combination of both, for overweight Latino adolescents (12–17). The purpose of this paper is to review the main and secondary outcomes from these intervention trials and to highlight which approaches or components of the interventions were successful at reducing obesity and type 2 diabetes risk factors in this very high-risk population.

## Description of the interventions

### Study 1: STEALTH (Strength Training Exercise for Adolescent Latinos To improve Health)

Twenty-one overweight Latino adolescent boys (15.1 ±± 0.5 years) were randomly assigned to either a strength-training group (ST; n=11) or a control group (n=10). A detailed description of the methods and findings have been previously reported (12). Participants in the ST group received strength training two times per week for approximately 60 minutes per session for 16 weeks. Training sessions took place on two non-consecutive days per week and the program was personalized and progressive such that the resistance increased as the participant's technique and strength improved.

### Study 2: ALAS (Adolescent Latinas Adjusting Sugar)

Twenty-three overweight Latina adolescent girls (14.5 ±±1.7 years) were randomized to a 12-week nutrition intervention delivered in either an individualized home-based format (n=11) or a group classroom-based format (n=12). A detailed description of the methods and findings have been previously reported (13,14). All participants received a 16-week culturally tailored, modified carbohydrate, dietary intervention (1x/wk), specifically focused on a reduction in added sugar and an increase in dietary fiber intake.

### Study 3: SANO LA (Strength and Nutrition Outcomes for Latino Adolescents)

Fifty-four participants (15.5 ±± 1.0 years, 52% male) were randomized to one of three groups: Control (C; n=16); Nutrition only (N: n=21); or Nutrition + Strength Training (N+ST; n=17). This intervention and the results have been described in detail elsewhere (15,16). Participants in the N group received a 16-week modified carbohydrate, dietary intervention (1x/wk; expanded from Study 2). Participants in the N+ST group received the same nutrition program, but also received strength training two times per week (similar to Study 1 protocol).

### Study 4: CAST (Combination of Aerobic and Strength Training)

As a supplement pilot study to the SANO study, an additional 15 female participants were randomly assigned to a Nutrition + Combination of Aerobic and Strength Training (N + CAST) group. A detailed description of the methods and results have been previously described (17). Participants in the N+CAST received the same nutrition education class (as listed above), and also received CAST exercise training two times per week for approximately 60 minutes per session. The CAST sessions were held on two non-consecutive days per week and included 30 minutes total of cardiovascular activity (e.g., treadmill, elliptical machines, and aerobic classes) coupled with 30 minutes total of strength training.

## Conclusions

Several components from the above interventions resulted in significant reductions in adiposity and type 2 diabetes risk factors in overweight Latino youth. Strength training improved insulin sensitivity (SI) by 45% in Latino boys, whereas the N, N+ST, N+CAST improved other indices of glucose control in Latino boys and girls. The CAST approach also resulted in significant reductions in all adiposity measures in Latina girls. The location of the nutrition intervention (home-based versus group classroom-based) did not affect health outcomes. Participants who decreased added sugar intake, increased dietary fiber and had increased parental attendance, regardless of intervention group, had significant improvements in insulin secretion, reductions in visceral adipose tissue, and improvements in insulin sensitivity and beta-cell function.

In general the pilot studies had a bigger impact on adiposity and metabolic parameters. Study 1 resulted in a big improvement in SI, but when this approach was combined with the nutrition group and tested in the larger RCT (i.e., Study 3), the SI effect was lost. Similarly, Study 2 resulted in significant reductions in BMI, but this effect was also lost in Study 3. This may be related, in part, to the smaller, more personalized nature of pilot studies and fewer staff interacting with participants. In addition, the pilot studies had simpler interventions and only targeted one behavior, which may have been easier for the participants to comply and follow.

However, ST and CAST approaches might be better suited for overweight populations. Obese children experience increased teasing during physical activities and sports (18), which may be a primary reason for reductions in their physical activity levels. Obese youth, especially those who are morbidly obese, may have difficulty exercising due to limitations in their balance skills and aerobic capacities, not to mention the self-esteem and self-confidence issues. Yet, research has shown that overweight children achieve greater scores during activity requiring muscular strength (19), and as a result may be more enthusiastic and compliant to programs that incorporate strength training. The CAST approach, which includes only short bouts of cardiovascular components coupled with strength training also allowed these obese teens to complete an accumulation of 30 minutes of cardiovascular exercise in an achievable fashion. The cardiovascular component in the CAST design was probably largely responsible for the reductions in adiposity.

The ST design resulted in significant improvements in insulin sensitivity in Latino boys (Study 1), while the ST and CAST approaches significantly improved glucose control in Latino boys and girls (Study 4). Recent studies have shown that strength training can increase insulin-mediated glucose uptake and enhance insulin sensitivity (20,21). Strength training improves up-regulation of components of the insulin-signalizing cascade, such as protein concentrations of the insulin receptor, protein kinase B, glycogen synthase, and GLUT-4 (21). These mechanisms support our findings that strength training improves insulin sensitivity and glucose control. It is well established that endurance cardiovascular exercise (both acute and chronic bouts) improves glucose tolerance, whole body insulin sensitivity and insulin action on skeletal muscle glucose transport in rodent models and adults (22,23). Increased insulin action on skeletal muscle glucose transport is associated with increased protein expression of glucose transporter GLUT-4 and insulin receptor substrate-1 (22,23). An increased dose of the CAST exercise approach (i.e., more than two days a week) may be needed to see an improvement on insulin sensitivity or action.

Independent of intervention group and energy intake, participants who made modest reductions in their sugar intake (∼1 soda a day) had significant reductions in insulin secretion (Study 2 and 3), and those who had fairly small increases in fiber intake (1/2 cup of beans a day) had significant reductions in visceral adipose tissue (Study 3). These findings parallel some of our previous cross-sectional analyses in over 200 Latino children (aged 9 to 17). We have previously shown that high intakes of total and added sugar, and sugary beverages were the only dietary components associated with adiposity and poor beta-cell function (24,25), while fiber intake was inversely associated with waist circumference and the metabolic syndrome (26). These results suggest that interventions using a modified carbohydrate approach, focusing on reductions in added sugar and increases in dietary fiber, could have huge impacts on reducing obesity and related diseases in Latino youth.

Although the exact mechanisms by which reductions in sugar intake and increases in fiber intake led to reductions in insulin secretion and visceral fat are unclear, there are several pathways to consider. Excessive dietary sugar intake can lead to a rapid rise in blood glucose and insulin levels. Hyperinsulinemia, in turn, down-regulates insulin receptors and leads to insulin resistance (27). The body then responds to increasing blood glucose by increasing insulin secretion. Thus, a habitual consumption of excessive sugar intake causes a vicious cycle of hyperinsulinemia, insulin resistance, and increased compensatory insulin secretion, which can lead to beta-cell dysfunction and eventually type 2 diabetes (28). Dietary fiber, particularly soluble fiber, has been shown to have an effect on lipid metabolism by binding to bile acids and mixed micelle components, such as free fatty acids, which can also lead to delayed intestinal uptake or increased fecal excretion of lipids (29). These effects reduce postprandial chylomicron triglyceride circulation, which can then lead to decreased lipid deposition, specifically in visceral adipose tissue (30). Collectively, these mechanisms support our findings that reducing sugar intake can have a direct impact on reducing insulin secretion and increasing fiber intake results in reductions in visceral adiposity.

In Study 3, parental attendance was positively related to increased insulin sensitivity and beta-cell function. Parental support, parenting styles, and the family environment have long been shown to be important components in the prevention and management of childhood obesity (31–33). Brownell et al. (32) found that obese adolescents who received a 16-week intervention where the parents and child were taught separately, lost more weight post intervention and were able to maintain it for a year compared with the intervention group in which the mother and child were taught together, or the child was taught alone. Although, parental support throughout an intervention has been successful at reducing child and adolescent obesity, little is known about the effects of parental involvement on metabolic parameters. In the current study, participants whose parents attended the most nutrition classes, taught separately, had the biggest improvements in type 2 diabetes risk factors. These findings, along with those from Brownell et al., also highlight the need to include a parental component, but that it may be best to teach parents separately from adolescents.

Some limitations of the studies should be noted. One potential limitation to consider relates to the small sample size of each study; however, this limitation is somewhat offset by use of precise measures of body composition (DXA), and glucose/insulin indices (OGTT and FSIVGTT), and control of various covariates. This particular sample is also relatively homogeneous, i.e., overweight Latino children, nevertheless, the homogeneity gives us the unique opportunity to conduct interventions to reduce type 2 diabetes risk factors, independent of potential ethnic variations in adiposity, or insulin and glucose dynamics in a very at risk population.

In summary, the smaller pilot studies that only targeted one behavior (diet or exercise) were more effective at reducing adiposity and type 2 diabetes risk factors in overweight Latino youth. However, there were various components of each of the interventions that were successful at reducing obesity and metabolic risk factors. ST in boys improved insulin sensitivity while the CAST improved glucose control and resulted in reductions in all adiposity measures in girls. Nutrition programs that focus on reductions in sugar and increases in fiber intake and include a parental component can have profound effects on reducing adiposity type 2 diabetes risk factors.
